# Phacoemulsification surgery: stepping into the mixed reality era

**DOI:** 10.1007/s10792-026-04164-x

**Published:** 2026-08-04

**Authors:** Maria Ludovica Ruggeri, Ruggero Tartaro, Alberto Quarta, Luca Vecchiarino, Rodolfo Mastropasqua

**Affiliations:** 1 Ophthalmology Clinic, Department of Neuroscience, Imaging and Clinical Sciences, University G. D’Annunzio Chieti-Pescara, Via dei Vestini 31, 66100 Chieti, Italy; 2 Eye Clinic, University Hospital of Chieti-Pescara, Via dei Vestini 31, 66100 Chieti, Italy

**Keywords:** Augmented reality, Mixed reality, Virtual reality, Cataract, Phacoemulsification, Intraocular lens

## Abstract

**Purpose:**

To test the applicability of a mixed reality headset in cataract surgery.

**Methods:**

Patients scheduled for phacoemulsification and intraocular lens implantation surgery were enrolled in our prospective interventional study. All patients underwent complete baseline ophthalmological examination and multimodal imaging analysis before surgery, which was scheduled and performed with the aid of the mixed reality headset. Primary outcomes were: surgical safety, efficacy and intraoperative parameters (Time lag, and surgical time duration). Secondary outcome was the occurrence of self-reported surgeon strain (SRSS), measured in a range from 0 to 5 and ergonomics, measured through the rapid upper limb assessment scale, RULA.

**Results:**

A total of 100 phacoemulsification surgeries were performed. Surgical success was recorded in all cases (100%) and no intraoperative complications were documented (0%). Mean time lag was 50 ms and the mean intraoperative surgery duration was 14 ± 6 min. The SRSS was graded as 0 in all cases with the absence of neck and back pain nor nausea or visual disturbance.

**Conclusions:**

The application of mixed reality in phacoemulsification surgery is an innovative, safe and effective tool in intraocular surgery.

**Supplementary Information:**

The online version contains supplementary material available at 10.1007/s10792-026-04164-x.

## Introduction

Recent advancements in Ophthalmology have significantly transformed this field, with meaningful changes in the areas of research, diagnostics, and therapeutics [1–3]. Over the years, groundbreaking innovations in medicine have also encompassed ophthalmic surgery, leading to the development of novel surgical techniques that offer improvements in ergonomics, educational value, and postoperative outcomes [4]. Despite the traditional ophthalmic surgery model, by relying on the presence of the microscope only itself, has achieved significant results in surgery establishing its effectiveness and safety, innovative models have made their steps into ophthalmic surgery and pioneering additional tools such as the heads up and three-dimensional (3D) systems, have thus achieved promising results in anterior and posterior segment procedures with significant ergonomics benefits.[5–7] As a result, these innovative techniques have enriched the landscape of intraocular surgery, offering a new way of performing successful procedures, which is today available mostly worldwide [8].

Overall, cataract surgery is one of the most commonly performed surgeries. Its estimates collect about 3.7 million cases per year in the United States [9]. The technique has always been improving for intraocular lens (IOL) materials, phacoemulsification techniques, surgical tools and visualization systems, being the hallmark of a world that is continuously moving on [10, 11].

Recently, our group of research has been exploring the potential applications of mixed reality visualization systems in Ophthalmic surgery [12, 13]. First analyzed in a pivotal study in intraocular surgery, the use of a mixed reality headset (MRH) demonstrated safety and efficacy in completing the procedure with overall patient, surgeon and learners satisfaction. In fact, the advantages of using this system not only rely on the continuous update during surgery and superimposing available systems, that allow the overlay of pre-operative data to the intraoperative scenario without interfering with the procedure, but also extends to the possibility of mentoring and discussing the surgery in the Operatory room (OR) and beyond, through the application of the headset related systems. [12, 13] Previously, Orione et al. have tested the applicability of the same device in ophthalmoplastic surgery, demonstrating its potential applications in the treatment of periocular areas and adnexa [14]. All this evidence supports the role of innovative tools in Ophthalmology; nevertheless, despite the striking impact they have, we must consider that to suit their full applicability, such models cannot exclude their potential in cataract surgery, which requires control, precision and effectiveness. Therefore, the aim of this study is to confirm the applicability of a MRH in cataract surgery.

## Materials and methods

Our prospective interventional study was approved by the Institutional Review Board (IRB) of the Gabriele d’Annunzio University, Chieti-Pescara, Italy (Code AR2024) and adhered to the tenets of the Declaration of Helsinki. The inclusion criteria were: patients aged 50–80 years scheduled for phacoemulsification and intraocular lens (IOL) implantation surgery, axial length between 21.00 and 25.00 mm ( used to exclude highly myopic or hyperopic eyes), nuclear sclerosis (grade ≥2 and/or cortical cataract) [15]. Exclusion criteria were: complicated cataracts (ig postuveitis, traumatic cataract/subluxated cataract, posterior polar cataract) axial length more than 25 mm/less than 21 mm, ophthalmological conditions other than the one addressed to surgery. Patients were enrolled among those accessing the Ophthalmology Clinic of Gabriele d’Annunzio University, Chieti-Pescara, Italy.

### Pre-operative examinations

All patients underwent complete baseline ophthalmological examination including best corrected visual acuity (BCVA), slit lamp biomicroscopy, intraocular pressure (IOP) measurement and fundus oculi examination. Additionally, participants were analyzed with Fundus autofluorescence, (FAF) Spectralis HRA+OCT (Heidelberg Engineering, Heidelberg, Germany) and true-color confocal fundus photography (iCare Eidon, Centervue, Padova, Italy), Spectral Domain OCT (SD-OCT) using Spectralis HRA+OCT (Heidelberg Engineering, Heidelberg, Germany), endothelial cell analysis through Nidek CEM-530 Specular Microscope (Nidek Technologies, Erlangen, Germany) and IOL power calculation using optical biometry (IOL Master 700; Carl Zeiss Meditec, Jena, Germany). The SD-OCT acquisition protocol included foveal 49 horizontal raster dense linear B-scans, and horizontal and vertical B-scans. Images were acquired by a dedicated technician and those with poor signal strength (< 25) were excluded and repeated.

### The surgery

All surgeries were performed by the same fellowship trained experienced surgeon (RM) using the ZEISS ARTEVO® Resight (Carl Zeiss Meditec, Jena, Germany) surgical microscope with the aid of the Apple Vision Pro (Apple Vision Pro, Apple Inc., Cupertino, CA, USA) MRH, taking care about preserving surgical sterility at all pre-operative and intraoperative stages.

As described in our previous analysis, the device was worn by the surgeon before scrubbing in to uneventfully preserve sterility during the procedures [12, 13].

To ensure the reduction of bias deriving from the fatigue assessment, no more than 5 surgeries were performed with the device everyday. The Apple Vision Pro is a MRH integrating dual micro-OLED displays, eye- and hand-tracking systems and real-time virtual content projection within the user visual field. During surgery, the device enables the surgeon to visualize preoperative imaging directly within the surgical field through an immersive mixed-reality interface, without interfering with the operative microscope visualization or surgical sterility.

Notably, the surgeon had previously performed a total of three phacoemulsification and IOL implantation surgeries with the same device, previously reported in our pilot study.[12] Before the surgery, the FAF, SD-OCT and IOL power calculation was superimposed to the surgical scenario, and a FaceTime call with one of the trainee out of the operatory room (OR) was started, to share the surgical steps, as reported in our tele-mentoring model [13]. All surgeries were performed using standardized surgical settings and phacoemulsification was therefore performed accordingly: Under topical anestesia, a 2.75 mm clear corneal incision and one side port incision were performed. Intracameral ophthalmic viscosurgical device (OVD) was injected and after capsulorhexis and hydrodelineation and hydrodissection, nuclear phacoemulsification was performed. Residual cortical material was removed using the irrigation/aspiration (I/A) probe. A foldable IOL was inserted, and after OVD removal, the corneal incisions were hydrated to ensure watertight closure. (Video 1) Topical antibiotics were administered after the procedure and prescribed to all patients. Follow-up examinations were scheduled at day 1, day 15 and day 30 after procedure.

### Outcomes

#### Primary outcomes

Primary outcomes were: surgical procedure efficacy and safety. Surgical efficacy was primarily defined as the achievement of the primary goal of the surgery, (ig phacoemulsification and IOL implantation). Additionally, intraoperative parameters were measured as primary outcomes: Time lag, surgical time duration. Time lag was assessed by calculating the frame difference between the physical movement and the corresponding movement displayed on the screen while recording the surgical activity on the MRH and the 3D monitor. All frames were independently reviewed by two examiners, and in cases of disagreement, a third examiner was consulted [13].

Surgical safety was measured as the occurrence of intraoperative complications (ig posterior capsular rate, presence of post-operative flare and anterior chamber cells), that, if present, were further characterized in terms of prevalence, type, time and severity. Postoperative BCVA and Endothelial cell density (ECD) was measured at all follow-up times.

Likewise, the collection of mean cumulative dissipated energy (CDE), ultrasound time and amount of fluid used during surgery were included for the purposes of the safety analysis.

#### Secondary outcomes

As secondary outcome, the presence of SRSS was evaluated by asking the surgeon to rate it in a scale from 0 to 5 and to further characterize it, whether present with an open-ended question for specifications. Furthermore, rapid upper limb assessment (RULA) was retrospectively observed to analyze the surgeon ergonomics during surgery. A standardized lateral video recording of the surgeon was obtained during the phacoemulsification phase. Recordings were independently assessed by two blinded ergonomic evaluators using the Rapid Upper Limb Assessment (RULA) tool. Mean RULA scores were subsequently analyzed for all procedures.

### Statistical analysis

Statistical analysis was performed using Jamovi software version 2.6.44. Continuous variables were reported as mean ± standard deviation (SD), whereas categorical variables were expressed as frequencies and percentages. Surgical efficacy and safety outcomes were analyzed descriptively. Intraoperative complications and adverse events were reported as percentage. For ergonomic assessment, mean SRSS and Rapid Upper Limb Assessment (RULA) scores were used. The RULA scores obtained by the two blinded ergonomic evaluators were averaged for analysis. Mean RULA score was descriptively analyzed. A *p*-value <0.05 was considered statistically significant. Assuming an expected surgical success rate of approximately 95%, a sample size of 100 eyes was calculated to provide a two-sided 95% confidence interval with a precision of approximately ±4–5%, which was considered adequate for our exploratory feasibility study.

## Results

A total of 100 phacoemulsification surgeries in 100 enrolled patients were performed in our prospective interventional study. Enrolled patients were aged 65 ± 7 years old, and 50 over 100 (50%) were female. Mean preoperative BCVA was 0.52 ± 0.18 logMAR and Mean ECD was 2350 ± 310 cells/mm^2^. Mean axial length was 22 ± 4 mm. Surgical success was recorded in all cases (100%) with a successful phacoemulsification and IOL implantation. Mean time lag was 50 ms and mean intraoperative surgery duration was 14 ± 6 min. No intraoperative (ig posterior capsular rupture, zonular dialysis, dropped nucleus or corneal burn) nor early or late post-operative surgical complications were recorded at all times. Mean cumulative dissipated energy (CDE) was 3.9 ± 1.5, mean ultrasound time was 6.0 ± 2.2 s, and mean fluid use was 75 ± 23 mL. Mean postoperative BCVA improved to 0.05 ± 0.08 logMAR, while mean postoperative ECD was 2338 ± 305 cells/mm^2^, showing no significant endothelial cell loss.The SRSS was graded as 0 with the absence of neck and back pain nor nausea or visual disturbance was signaled while the ergonomic assessment through RULA demonstrated a mean score of 2.8 ± 0.6 (Table 1).

**Table 1:** Study design. Baseline demographics, primary and secondary outcomes are reported

	n = 100
*Baseline demographics*	
Female, *percentage*	50
Age, *years old*	65 ± 7
Preoperative BCVA, *LogMAR*	0.52 ± 0.18
Preoperative ECD, *cells/mm*^*2*^	2350 ± 310
Axial Lenght, *mm*	22 ± 4
*Primary outcomes*	
Surgical success, *percentage*	100
Time lag, *ms*	50
Surgical time duration, *minutes*	14 ± 6
Surgical safety, *percentage*	100
Postoperative BCVA, *LogMAR*	0.05 ± 0.08
Postoperative ECD, cells/mm^2^	2338 ± 305
*Secondary outcomes*	
SRSS, *mean*	0
RULA, *mean*	2.8 ± 0.6

Values are reported in mean ± standard deviation

## Discussion

The worldwide high surgical rate of cataract surgery imposes the necessity of validating innovative systems in this surgery, acknowledging the necessity to develop useful systems applicable to the daily Ophthalmology practice. In fact, while innovation enriches this field by developing advanced systems that offer an immersive experience to the user, the validation of a model must encounter the characteristics of practicability and easy performance. In this light, while already reported in our pilot study, and further described in our tele-mentoring model, the present study aimed to confirm the feasibility of a MRH in cataract surgery [12].

While previous studies have validated the 3d and heads-up systems (HUS) in phacoemulsification surgery, enhancing the advantages also in terms of ergonomics, including the reduced incidence of neck and back pain among surgeons, none of them has specifically focused on the potential application of the MRH in this field [5, 16]. In fact, to our knowledge little is known about the potential applicability of this MRH in performing successful phacoemulsification and IOL implantation procedures [12, 14]. In our pilot study, the low number of cases and the absence of intraoperative parameters prevented us from drawing conclusions regarding the significant application of this headset in performing cataract surgery. As a result, the present analysis is intended as a larger confirmatory study to confirm the feasibility of the use of the MRH in cataract surgery, by obtaining successful results in all enrolled cases with IOL implantation and no intraoperative complications recorded at all follow-up times, alongside acceptable intraoperative surgical time duration and time-lag [17].

Of note, we did not experience any device-related complications in our cases, which can anyway potentially occur, such as limited battery life and whose impact in the surgical flow should be further assessed in future studies.

With acceptable results in surgical duration time and time lag, our analysis sheds light on possible improvements that may derive from the application of this methodology to routine surgical treatments [18–20]. Specifically, real-time hands-free intraoperative access to multimodal preoperative data with no sterility alteration, enhanced spatial integration of imaging within the surgical workflow, improved remote interaction and educational capabilities, alongside potential ergonomic benefits for the surgeon, were found. In fact, in our cases the surgeon did not experience any ergonomical nor visual disturbance thus evidencing the absence of surgeon physical stress related to the surgery, in accordance with previous reports on 3d and HUS systems and our preliminary analysis on the use of the device [12, 21].

However, our feasibility study did not aim at a comparison with the currently available systems, and it must be acknowledged that it focused on a regular daily volume of cataract surgery, while future studies should assess the performance of the MRH in a high-volume surgical day.

In this analysis, the MRH has offered the surgeon the possibility of overlaying the pre-operative analysis to the surgical field with any disturbance, despite the necessity of further studies aimed at comparing this innovative approach with the traditional microscope-based cataract surgery. Nevertheless, the implementation of this innovative platform opens the surgery to other benefits that may be derived from its use. In fact, while the potential surgical tele-mentoring advantages have already been addressed by our previous analysis in vitreoretinal training, its application may be extended to phacoemulsification surgery as well, due to the ease in communicating with the close and far presence of clinicians and/or trainees. (XX).

According to the World Health Organization, despite cataract being the most commonly performed surgery, a certain amount of worldwide blindness is still attributable to its prevalence. Specifically,

recent reports have evidenced that 46.53% of global blindness is today attributable to cataract, making it one of the leading causes of treatable blindness. [22]

In this light, the possibility of sharing knowledge through the Facetime platform extends its teaching benefits, by training young surgeons in cataract surgery even at a certain distance, despite the necessity of a stable internet connection that may be difficult to achieve in some rural settings potentially creating a barrier for widespread adoption. Nevertheless, future studies should be addressed to fill this gap, to reduce the level of present blindness due to cataract.

Furthermore, potential advantages may derive from its application in cornea and refractive procedures.

In terms of intraoperative advantages, the opportunity of superimposing the acquired multimodal imaging to the surgical field with no disturbance exploits its benefits in phacoemulsification while confirming the final refraction, and in any case of intraoperative complications that may require the use of a different IOL than the one previously discussed, allowing the surgeon to observe directly the pre-operative examination while preserving sterility. Likewise, although preoperative patient assessment remains a cornerstone of surgical success, the possibility of reviewing structural imaging during surgery introduces opportunities for intraoperative reassessment and potential modification of the surgical trajectory in certain procedures.

Besides, one should discuss that while the headset has demonstrated to be successful in Vitreoretinal surgery, the applicability of the Apple Vision Pro MRH in cataract surgery enables the possibility of performing combined surgery with the MRH, exploiting the advantages already exposed.

However, our analysis has limitations. First, the study design- single surgeon, single-center analysis with the absence of a control group prevented us from comparing it with existing systems. Secondly, the presence of self-reported measurements and potential bias deriving from inclusion criteria such as the inclusion of only uncomplicated cataracts. Furthermore, long-term safety should be assessed including the occurrence of post-operative complications beyond day 30.

While future studies should investigate the specific comparison between the current systems ( including traditional microscope-based surgery and 3D surgery) and the MRH guided surgery in phacoemulsification, our analysis explores the validation and applicability of a MRH in the largest cohort of patients undergoing cataract surgery, with this innovative headset, further confirmed by the compliance of intraoperative and postoperative parameters with encouraging results in safety and efficacy.

While one should rely on validated traditional microscope-based surgery, attention should be given to innovation and new systems, acknowledging both limitations and advantages and allowing phacoemulsification to step into the mixed reality era.

## Supplementary Information

Below is the link to the electronic supplementary material.

Supplementary file1: Video 1. The application of the mixed reality headset in Phacoemulsification surgery. The video shows the application of the mixed reality headset in a case of phacoemulsification surgery. Once in the Operatory Room, the surgeon easily navigates in evaluating the multimodal imaging of the surgical case analyzing the pre-operative exams without interfering with the surgical field and his activity, which continues uninterruptedly

## Data Availability

No datasets were generated or analysed during the current study.
